# Geospatial Analysis of Mass-Wasting Susceptibility of Four Small Catchments in Mountainous Area of Miyun County, Beijing

**DOI:** 10.3390/ijerph16152801

**Published:** 2019-08-06

**Authors:** Chen Cao, Jianping Chen, Wen Zhang, Peihua Xu, Lianjing Zheng, Chun Zhu

**Affiliations:** 1 College of Construction Engineering, Jilin University, Changchun, Jilin 130026, China; 2 Department of Architectural Engineering, Changchun Sci-Tech University, Changchun, Jilin 130600, China; 3 State Key Laboratory for Geomechanic and Deep Underground Engineering, China University of Mining and Technology, Beijing 100083, China

**Keywords:** mass-wasting susceptibility, catchment management, frequency ratio, information value, farmland terraces

## Abstract

Driven by the pull of gravity, mass-wasting comprises all of the sedimentary processes related to remobilization of sediments deposited on slopes, including creep, sliding, slumping, flow, and fall. It is vital to conduct mass-wasting susceptibility mapping, with the aim of providing decision makers with management advice. The current study presents two individual data mining methods—the frequency ratio (FR) and information value model (IVM) methods—to map mass-wasting susceptibility in four catchments in Miyun County, Beijing, China. To achieve this goal, nine influence factors and a mass-wasting inventory map were used and produced, respectively. In this study, 71 mass-wasting locations were investigated in the field. Of these hazard locations, 70% of them were randomly selected to build the model, and the remaining 30% of the hazard locations were used for validation. Finally, a receiver operating characteristic (ROC) curve was used to assess the mass-wasting susceptibility maps produced by the above-mentioned models. Results show that the FR had a higher concordance and spatial differentiation, with respective values of 0.902 (area under the success rate) and 0.883 (area under the prediction rate), while the IVM had lower values of 0.865 (area under the success rate) and 0.855 (area under the prediction rate). Both proposed methodologies are useful for general planning and evaluation purposes, and they are shown to be reasonable models. Slopes of 6–21° were the most common thresholds that controlled occurrence of mass-wasting. Farmland terraces were mainly composed of gravel, mud, and clay, which are more prone to mass-wasting. Mass-wasting susceptibility mapping is feasible and potentially highly valuable. It could provide useful information in support of environmental health policies.

## 1. Introduction

Mass-wasting is a common occurrence throughout anthropogenic development [1]. Mass-wasting is a natural phenomenon by which rock, soil, or debris move downwards due to the action of gravity. It describes all of the processes that act continuously with varied intensity on all types of slopes to lower the ground surface. The mass-wasting process is controlled by the interaction of geological agents and processes with the geo-materials. The degree and type of movements depend upon a few aspects of geology, environment, geomorphology, hydrology, and some additional environmental stress factors, including biotic factors. Thus, the extent of mass-wasting damage is extensive. Mass-wasting is related to hazards caused by gravity, such as landslides, collapses, and debris flow. Mass-wasting maps are a very important component of catchment management.

Anthropogenic activities, including farmland expansion and timber harvesting, change natural conditions, which increases the risk of mass-wasting occurrence. Although mass-wasting hazards are inevitable, it is important to identify areas in which mass-wasting events are likely to occur [2]. Thus, land use managers should be able to identify all aspects of landscape vulnerability [3]. Mass-wasting susceptibility mapping has been recognized as the first necessary step in hazard prevention and its management [4].

Remote sensing (RS), geographic information systems (GIS), and Global Positioning Systems (GPS) are now widely applied as so-called “3S technology”. RS and GIS techniques have been applied for different mass-wasting hazard susceptibility models [5,6], and appropriate assessment methods should be similarly applied for mass-wasting susceptibility mapping [7,8,9]. The use of GIS has greatly progressed in the field of environmental science in applications such as landslide and groundwater susceptibility mapping and flash flood hazard susceptibility mapping. These technologies can provide a good perspective for mass-wasting assessment research. Various landslide susceptibility maps, debris flow susceptibility maps, and rockfall susceptibility maps have been created in different countries [10,11]. The use of RS and GIS has increased significantly in response to the need for rapid data collection and improved mass-wasting bitmaps for commercial satellite products. Further, GIS is a useful tool for studying events with multidimensional behavior; for example, mass-wasting hazards are investigated using a variety of spatial–temporal models. In order to obtain accurate results from these models, it is vital that the input factors retain their spatial associations [4].

Various technologies involving GIS and RS have been developed by researchers in the field of environmental science. Among the different approaches, natural hazard zoning has been applied to the most popular and widely used models, including weights of evidence [12,13,14], Shannon’s entropy [15], random forest [16,17,18], logistic regression [19,20,21,22], statistical index [23,24], and analytical hierarchy process [25,26]. Nowadays, machine learning algorithms, including artificial neural networks [27,28,29,30], support vector machines [31], and decision trees [32], are also widely used. Frequency ratio (FR) and information value (IV) models have already been frequently used for landslide susceptibility mapping [33,34]. Similar models have been used in gully erosion [35], landslide susceptibility mapping [36,37], and forest fire susceptibility [38,39]. The FR method has been proven to be effective, and it has been successfully applied to flash flood hazard susceptibility mapping and landslide susceptibility mapping [24,40,41]. In view of the effectiveness of the FR method, it was selected as a statistical method in the present study to better explore the effect of different mapping units on the susceptibility mapping of debris flow. Furthermore, the information value model, which has been proven to be a very useful method for measuring the degree of influence of each causative factor, is a simple probabilistic bivariate statistical method whose accuracy is acceptable [42,43].

Farmland terraces are abundant in catchments, and their structural strengths are very low. Small landslides often form, and large numbers of terraces have a high risk of being damaged. Thus, it is very important to highlight protection and adaptation approaches for agricultural areas to minimize the consequences of mass-wasting due to different human activities and climate change conditions. On the other hand, technical measures, such as farmland terraces, can be used for soil and water conservation. To a certain extent, these terraces could be an approach to intercepting an oncoming hazardous event; in turn, the terraces would sustain damages. Terrace stone walls have been reconstructed by local residents when erosions or landslides have occurred in the past. Thus, field surveys have found that most farmland terraces have been well maintained. Studies have been conducted in middle and low farmland areas to establish relationships between farmland maintenance and rainfall on a sub-catchment scale. Because forests can guarantee less soil erosion and keep the slope more stable [44], protecting forestland is imperative. Farmland terraces, which can retain water in catchments, also contribute to the alleviation of mass-wasting. Since farmland terraces also suffer damages from different conditions, local residents should pay more attention to them. Considering that there are many farmland terraces in mountainous areas in Beijing, especially the intermediate- and low-elevation areas, appropriate mass-wasting management plans for these areas are vital.

This study aims to determine the spatial probability of mass-wasting occurrence in four catchments. The correlation between influencing factors and mass-wasting inventory is identified, and the accuracy is evaluated. Furthermore, the present work also conducts a comparative assessment of two statistical models used for mass-wasting susceptibility mapping: the frequency ratio (FR) model and information value model (IVM). The FR and IVM methods were selected for their mathematical simplicity, their ability to extract data in a limited time period, and their effectiveness. The prediction accuracy and performance of each method were assessed using four catchments in a mountainous area. Nine mass-wasting susceptibility factors were used in the two models using GIS software. The results were validated using the area under the receiver operating characteristic curve (ROC) method.

## 2. Study area and Inventory Maps

**Figure 1:**
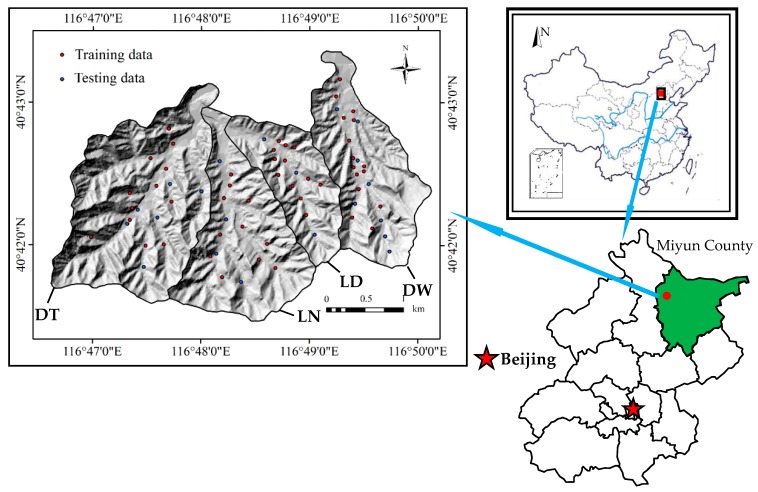
Geographical position of the study area. Note: DT = Duitaizi catchment; LN = Lamazhazi Nan catchment; LD = Lamazhazi Dong catchment; DW = Dawa catchment.

The study area is situated in the west of Miyun County, northeast of Beijing. This area contains four catchments (Figure 1), namely, Dawa (DW), Lanmadonggou (LD), Lamanangou (LN), and Duitaizi (DT). The area is located from 116°46’21” to 116°50’04” E longitude and from 40°41’17” to 40°43’14” N latitude, covering an area of 10.19 km^2^. The areas of the four catchments are 2.239, 1.484, 2.587, and 3.88 km^2^, respectively. The study area is dominated by hills and the elevation ranges from about 610 to 1280 m above sea level. The average annual temperature is 10.8 °C, and the annual rainfall is approximately 661.3 mm. The lithology of this area is dominated by gneiss (A_rsu_^1^), quartzite (A_rsa_^3^), diorite (δ_5_^2^), acid rock (γ_5_^2^), and granite (γ_5_^3^). The terrain of Miyun County is primarily mountains and hills, and plains are only distributed in the southwest of the area. Mountains account for 46.7% of the total area, hills account for 36.6%, plains account for about 8.3%, and in the middle of the area, the Miyun reservoir accounts for about 8.4%.

### 2.1. Identifying Locations of Mass-Wasting Inventory

The future mass-wasting can be estimated by analyzing past records. An inventory map can show the distribution and characteristics of mass-wasting in the study area [19]. Mass-wasting events come in many shapes, sizes, and speeds. Typically, the steeper the angle of a slope, the faster the down-slope movement of rock and sediment. Also, water can play a significant role in mass-wasting, sometimes acting as the key component to a mass-wasting event, or serving as a lubricant within a mass of sediment and rock, enabling it to travel faster and further than it would otherwise. Types of mass-wasting mainly contain rock fall and rock avalanche, rock slide and slump, debris flow, earth flow, and creep.

**Figure 2:**
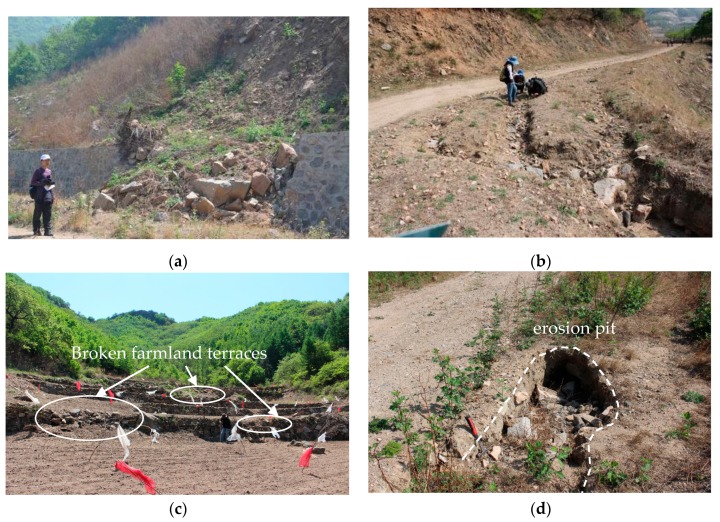
Mass-wasting in the field: (**a**) broken retaining wall; (**b**) erosion gully; (**c**) broken farmland terraces; and (**d**) erosion pit.

**Figure 3:**
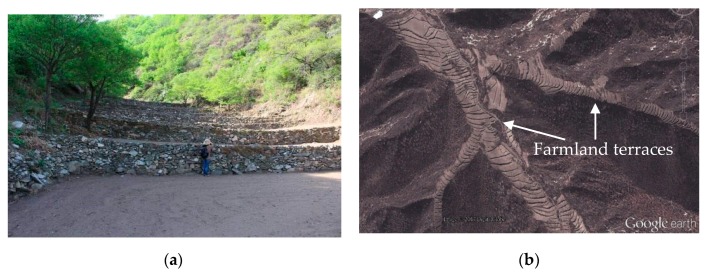
Farmland terraces in the study area: (**a**) field photo; (**b**) image from Google Earth.

The mapping of mass-wasting in the four catchments is necessary to depict the relationship between susceptibility ranges and influencing factors. Extensive field investigation and observations were conducted to produce a comprehensive and reliable inventory map. The mass-wasting inventory map shows the spatial distribution of mass-wasting in the study area. This was used as a base map to generate the mass-wasting susceptibility map. We analyzed records of mass-wasting to identify susceptible areas that were prone to occurrence of new mass-wasting (Figure 2). The inventory map was first created by locating mass-wasting in the four catchments using documents and detailed field surveys. A good source of information includes interviews with local residents, which were conducted to identify destroyed houses and public facilities damaged by mass-wasting that occurred before. The storm and flash flood on 21 July 2012, left local residents with significant impressions, giving this event special attention. Field surveys confirmed landslides, collapses, and erosion, which were regarded as mass-wasting. The farmland terraces are mainly located at the bottom of the catchment and partly on the hillside (Figure 3), so they are highly vulnerable to flash flooding or debris flow and are easily damaged. Identifying the locations of mass-wasting is fairly straightforward.

From an inventory map, a mass-wasting susceptibility map can be produced. A mass-wasting susceptibility map was generated using a previous inventory map and remote sensing images. Seventy-one mass-wasting locations were surveyed in the four catchments and were used in further analysis. Fifty mass-wasting locations were randomly selected to build and train the models. The remaining 21 mass-wasting locations were used as validation data.

### 2.2. Influence Factors

Various factors, such as a heavy storm, geographic and geomorphic conditions, and human activities, were deemed to be the main conditions causing mass-wasting. Various thematic data layers, including elevation, slope angle, plan curvature, stream power index (SPI), topographic wetness index (TWI), lithology, land use, soil type, and flow accumulation, were prepared. In the development of a model for evaluation of mass-wasting-susceptible areas, it is crucial to identify practical, reasonable, and easily obtained influencing factors. The above-mentioned factors were selected because they have been successfully used in previous work. The original data used in this study are shown in Table 1.

**Figure 4:**
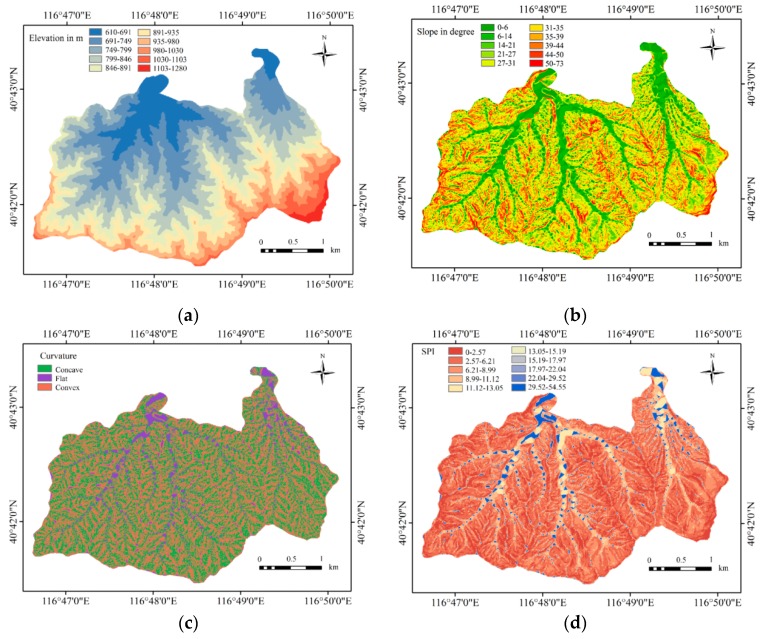

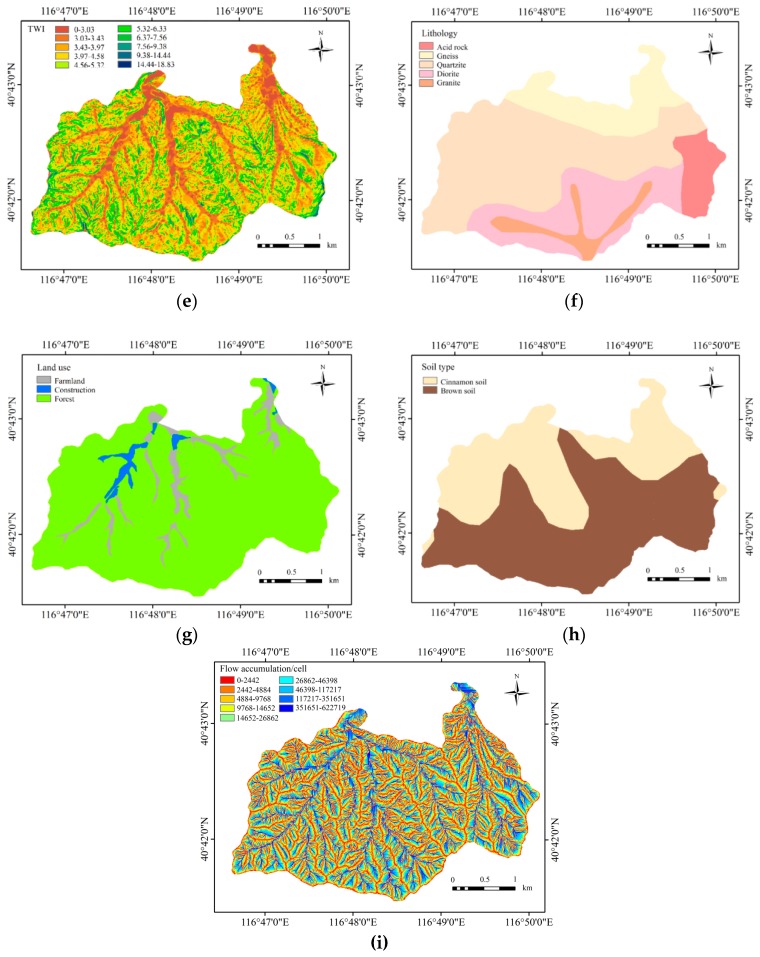
Input thematic layers: (**a**) elevation; (**b**) slope; (**c**) curvature; (**d**) stream power index (SPI); (**e**) topographic wetness index (TWI); (**f**) lithology; (**g**) land use; (**h**) soil type; and (**i**) flow accumulation.

A topographic map with a scale of 1:10,000 was used to produce a digital elevation model (DEM) with a resolution of 5 m. The maps for four factors—slope angle, plan curvature, SPI, and TWI—were produced from the DEM using GIS software. Of the nine factors, elevation, slope angle, SPI, and TWI were categorized using the natural break method. Cao et al. [24] proved that using the natural break method [45,46,47] is more appropriate than using manual classification to categorize factors. In the study area, the elevation varies between 610 and 1280 m. Figure 4a shows the elevation map of the study area.

Elevation was divided into ten classifications using the natural break method: (1) 610–691 m, (2) 691–749 m, (3) 749–799 m, (4) 799–846 m, (5) 846–891 m, (6) 891–935m, (7) 935–980 m, (8) 980–1030 m, (9) 1030–1103 m, and (10) 1103–1280 m. The slope angle is an important factor because it is easier for water to infiltrate the soil in flat areas [48], and the surface runoff and water velocity are controlled by the slope angle. The slope angle map was taken from the digital elevation model with 5 × 5 m raster cells. The slope angle was divided into ten classifications using the natural break method (Figure 4b): (1) 0–6°, (2) 6–14°, (3) 14–21°, (4) 21–27°, (5) 27–31°, (6) 31–35°, (7) 35–39°, (8) 39–44°, (9) 44–50°, and (10) 50–73°. Negative plan curvature describes concavity, zero plan curvature indicates flatness, and positive plan curvature defines convexity (Figure 4c). The stream power index (SPI) is the power of the water flow in cases of erosion [49]. The SPI map is shown in Figure 4d. The topographic wetness index (TWI) defines the amount of water flow accumulated at any point in a catchment and the ability of the water to flow downward under gravity [50]. The TWI map (Figure 4e) was prepared and divided into ten subclasses using the natural break method. The SPI and TWI are defined as

(1) $$\mathrm{SPI}=A_{s}\tan\beta$$

(2) $$\mathrm{TWI}=\ln(A_{s}/\tan\beta )$$

where *A_s_* is the specific catchment area (m^2^/m) and *β* (radians) is the slope (in degrees) [51].

The types of lithology were taken from a geological map with a scale of 1:10,000 (Figure 4f). Five lithology subclasses were used: acid rock (γ_5_^2^), gneiss (A_rsu_^1^), quartzite (A_rsa_^3^), diorite (δ_5_^2^), and granite (γ_5_^3^). The land use data were acquired using a Google Earth image from 3 May 2014. The land use data were verified by field investigation and remote sensing interpretation, and the land uses were defined as farmland, construction areas, and forest (Figure 4g). The land use type influences infiltration, water convergence, and the relationship between the surface water and groundwater. Different vegetation types have different capacities of rainfall interception and water storage. The type of vegetation also affects the time and size of water confluence. Figure 4h presents the soil types, which are cinnamon soil (40.88%) and brown soil (59.12%) in this study area. The soil type determines the water infiltration, and it controls surface runoff and submergence processes. This study applied flow accumulation as an influence factor. The basic idea is that the DEM represented by regular grids has a unit of water at each point. Natural water flows from a high point to a low point, and the amount of water that flows through each point depends on the flow direction. The convergence of each grid shows the flow accumulation and reflects the amount of water in each grid in the area. The flow accumulation map is shown in Figure 4i. Flow accumulation was categorized into nine subclasses: (1) 0–2442, (2) 2442–4884, (3) 4884–9768, (4) 9768–14,652, (5) 14,652–26,862, (6) 26,862–46,398, (7) 46,398–117,217, (8) 117,217–351,651, and (9) 351,651–622,719.

## 3. Methodology

### 3.1. Frequency Ratio

The FR method is an accurate and effective technique that is based on the observed relationships between the distribution of debris flows and related factors. In this study, the FR method was used to perform mass-wasting susceptibility mapping. The FR is defined as the ratio of the probability of the occurrence of a mass-wasting to the probability of a nonoccurrence for a given attribute [52,53]. The larger the FR, the stronger the effect of the given factor on the debris flow [54]. This approach reveals the correlation between mass-wasting susceptibility areas and the influence factors in the catchment. First, the FR for each factor type or range was calculated using Equation (3):

(3) $$FR=\frac{C/D}{M/N}$$

where *C* is the number of cells with mass-wasting in each influencing factor subclass; *D* is the total number of cells with mass-wasting in the four catchments; *M* is the cell number of each influencing factor subclass; *N* is the total cell number of the four catchments. FR values greater than 1 indicate higher densities of mass-wasting in the category compared with the density of hazards in the four catchments, and these translate to a higher correlation between the category and the occurrence of mass-wasting. FR values less than 1 indicate a lower correlation [55]. The mass-wasting susceptibility index (MW*SI*) was calculated using Equation (4):

(4) $$MWSI=\sum_{i=1}^{N}FR$$

where *FR* is the weight of the *FR* model, and *N* is the number of influencing factors. The greater the *MWSI*, the higher the possibility that mass-wasting will occur.

### 3.2. Information Value Model

The information value model (IVM) is a quantitative analysis method developed from information theory. The information value method is a bivariate statistical approach to deriving data for mass-wasting areas, as well as the unaffected areas. With this method, the probability of mass-wasting occurrence in the study area can be quantified in the mass-wasting classes. Yin and Yan [56] proposed this method, and Van Westen [57] modified it. It involves the computation of (1) the cell number of total mass-wasting for each influence factor subclass and (2) the cell number of total pixels of mass-wasting in the study area. Recently, this method has become increasingly favored by scholars and has been applied to geological hazard assessment and environmental evaluation [58,59,60].

The information value *I* (*x_i_*, *H*) of each influencing factor *x_i_* is

(5) $$I(x_{i},H)=\ln\frac{N_{i}/N}{S_{i}/S}$$

where *N_i_* is the number of cells with mass-wasting in each influencing factor subclass *x_i_*, *N* is the total number of mass-wasting in the study area, *S_i_* is the area of each influencing factor subclass *x_i_*, and *S* is the total number of cells in the four catchments. The information value of each influencing factor subclass is calculated as

(6) $$I_{i}=\sum_{i=1}^{n}I(x_{i},H)=\sum_{i=1}^{n}\ln\frac{N_{i}/N}{S_{i}/S}$$

where *I_i_* is the total information value of each influencing factor subclass, and *n* is the number of influencing factor subclasses.

**Figure 5:**
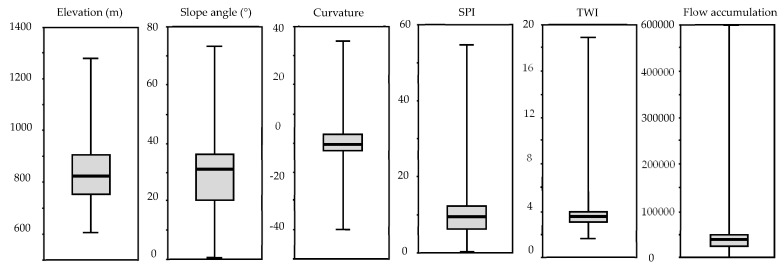
Boxplots showing the distribution of six influence factors in the study area: elevation, slope angle, curvature, SPI, TWI, and flow accumulation.

**Figure 6:**
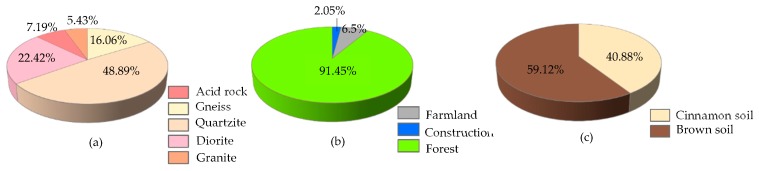
Subclass ratios of three influence factors: (**a**) lithology; (**b**) land use; (**c**) soil type.

Figure 5 and Figure 6 summarize the distribution of the nine influence factors.

## 4. Results

### 4.1. Mass-Wasting Susceptibility Mapping Using the FR Model

Table 2 shows the results for the spatial relationship of the mass-wasting susceptibility area and accommodation factors from the frequency ratio model. The results using the FR method show that mass-wasting is mostly located at elevations of 691–846 m. The elevation subclass 691–749 m has the highest FR value of 2.152. In the high-elevation region, the occurrence of hazards is very low. Botzen [61] proved that mass-wasting does not easily occur in high-elevation regions. The slope angle subclass 0–21° has a high FR value, while a slope angle higher than 50° has a low likelihood of mass-wasting occurrence. The highest FR value is 2.622 in the 14–21° subclass, followed by subclasses 6–14° and 0–6°, which have FR values of 1.66 and 1.57, respectively. Analysis of the frequency ratio between mass-wasting and plan curvature shows that the flat subclass has the highest FR value of 1.731, and the concave subclass has a value of 1.356. From the SPI results, the highest frequency ratio is related to the subclass 8.99–11.12 (2.989), followed by the subclass 15.19–17.97 (2.482). The FR values for the TWI show that the subclass 3.03–3.43 has the highest value of 2.561. The highest FR value for lithology is 1.619 in gneiss. The FR values for the remaining lithology elements, including quartzite, diorite, and acid rock, are 1.064, 0.892, and 0.278, respectively. The FR value of farmland is 1.846. Farmland is prone to occurrence of hazards. The FR value for forest areas is 0.962. In terms of soil type, the FR values of cinnamon soil and brown soil were 1.125 and 0.914, respectively. Higher flow accumulation was correlated with higher FR values, i.e., 3.382 (351,651–622,719 subclass) and 1.458 (117,217–351,651 subclass).

**Figure 7:**
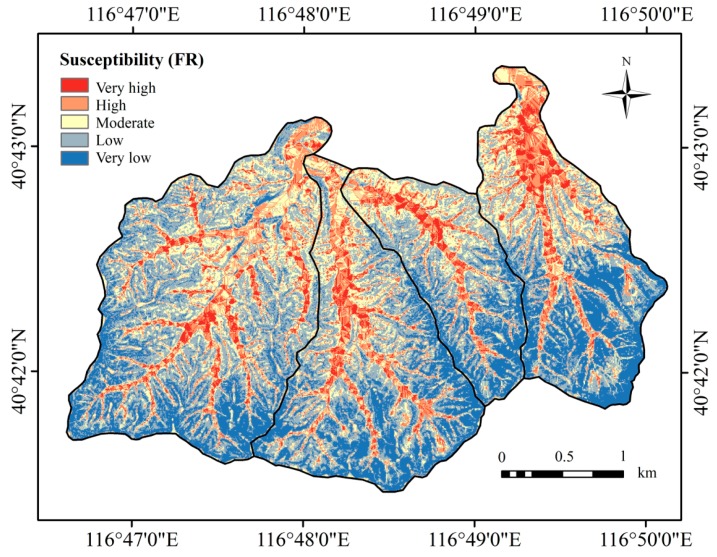
Mass-wasting susceptibility map generated by using the frequency ratio model.

Figure 7 shows the FR mass-wasting susceptibility mapping results. The five hazard susceptibility classes are very low (3.3–6.9), low (6.9–8.87), moderate (8.87–11.23), high (11.23–13.92), and very high (13.92–20.02). The areas corresponding to the five classes are 2.5, 3.34, 2.24, 1.41, and 0.7 km^2^ (Table 3). Thus, the FR model shows that 20.71% of the study area is highly susceptible to mass-wasting hazards.

### 4.2. Mass-Wasting Susceptibility Mapping Using the IV Model

The calculated information value of each class of mass-wasting influencing factor is shown in Table 2. For elevation, most mass-wasting hazards occur between 691 and 846 m. The maximum information value (IV) was found in the elevation subclass 691–749 m (0.766). This area has the highest possibility of occurrence of mass-wasting hazards. In terms of the slope angle, the highest probability of mass-wasting hazard occurrence is found in the range of 14–21° (0.964), followed by 6–14° (0.507) and 0–6° (0.451). For plan curvature, flat and concave areas have higher IVs of 0.549 and 0.304, respectively. According to the application of the IV model, the SPI subclass 8.99–11.12 has a higher IV (1.095). The subclass 15.19–17.97 is also prone to hazards. In the case of the TWI, most of the mass-wasting occurs in the subclasses 3.03–3.43 (0.94), 0–3.03 (0.273), and 7.56–9.38 (0.223). For the lithology factors, the highest IV value is 0.482 for gneiss. The IVs of the other lithology factors, including quartzite, diorite, and acid rock, are 0.062, −0.0114, and −1.127, respectively. The IV of farmland is 0.613, which is the only positive value for the land use influencing factor. Farmland is prone to hazard exposure. The IV values of forest and construction land are −0.038 and −1, respectively. In terms of soil type, the IV values of cinnamon soil and brown soil are 0.118 and −0.091, respectively. Higher flow accumulation is correlated with higher IVs, i.e., 1.218 (351,651–622,719 subclass) and 0.377 (117,217–351,651 subclass).

**Figure 8:**
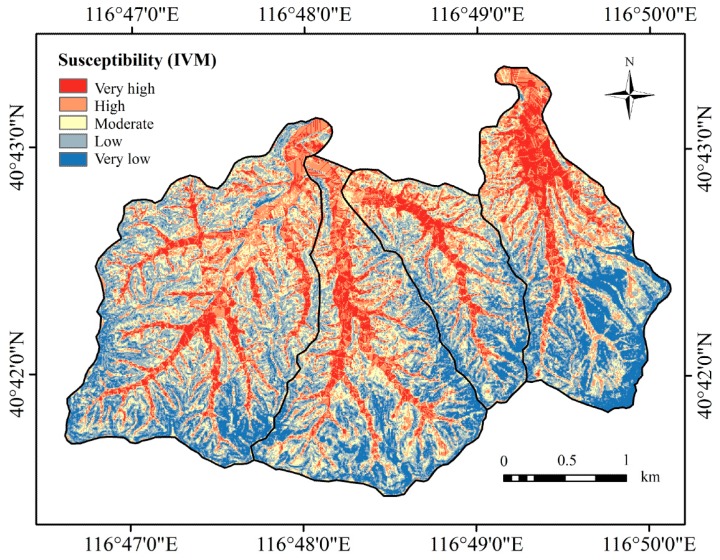
Mass-wasting susceptibility map generated by using the information value model.

Figure 8 shows the mass-wasting susceptibility map generated by using the IVM. The map is divided into five grades: very low (−7.3 to −3.67), low (−3.67 to −1.74), moderate (−1.74 to 0.19), high (0.19–2.23), and very high (12.23–6.75). The areas of the five classes are 1.76, 2.78, 2.40, 2.01, and 1.24 km^2^ (Table 3). Thus, the IV model shows that 31.87% of the study area is highly susceptible to mass-wasting hazards.

Table 2 summarizes the results of the FR and IV models for each identified class. A lower elevation region easily accumulates water, agreeing with the results calculated by the FR and IV models. The results for the slope angle show that the maximum frequency corresponds to subclasses with lower slope angles; this is because of rainfall water accumulating in these areas. Flat plan curvature has the highest value according to both the FR and IV models, highlighting the high possibility of mass-wasting hazards in relatively flat and concave areas.

### 4.3. Validation of Mass-Wasting Susceptibility Mapping

**Figure 9:**
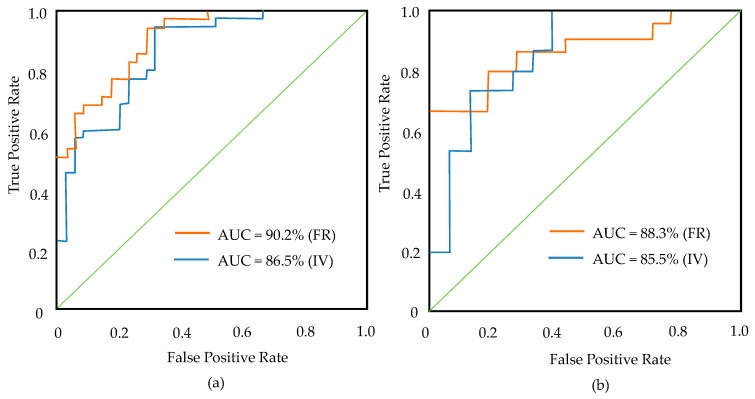
Success and prediction rate curves using the frequency ratio and information value methods: (**a**) success rate curve and (**b**) prediction rate curve.

Validation of both models was performed using the area under the receiver operating characteristic (ROC) method, which assesses the predictive power of a model and has been used in many works [35,62,63,64,65]. Validation of the mass-wasting susceptibility results is one of the most important tasks [66]. In this study, the results of mass-wasting susceptibility mapping were validated by the receiver operating characteristic (ROC) technique. In the ROC curve, the vertical axis represents the true positive rate, and the horizontal axis represents a false positive rate. The area under the curve (AUC) was used to evaluate the validity of the four models. From the training and testing data, the success and prediction rates of six models were calculated by using the AUC. The value of the AUC varies from 0.5 to 1, and the accuracy of the model is high if the value of the AUC is close to 1. Figure 9 shows the accuracy and prediction ability of the two models, which are assessed by comparing the success and prediction rates. The AUC values for the FR and IV models are measured by their success rate curves of 0.902 and 0.865, respectively, which reflect accuracies of 0.902 and 0.865 for the two models. The prediction rate curves show that the AUC values are 0.883 (FR) and 0.855 (IV). The quantitative relationship between the AUC and the model prediction accuracy is divided into the following grades: 0.5–0.6 is weak, 0.6–0.7 is moderate, 0.7–0.8 is good, 0.8–0.9 is very good, and 0.9–1 is excellent [64]. Both methods have a high success rate (0.8–0.9) and prediction rate (0.8–0.9). Therefore, the two methods perform very well when applied to mass-wasting susceptibility mapping.

## 5. Discussion

The slopes of the main channels are mostly large. The impact of a high intensity rainfall is strong enough to destroy vegetation, roads, farmland terraces, etc. Runoffs carry a large amount of silt, resulting in siltation and erosion. However, compared with debris flow, the destructive power of flash flooding is relatively weaker. The bark near the tree roots has been moderately damaged in the main channel of the DW catchment. The erosion marks all face the sourcing area and have a length of 0.5–1.2 m. The width of the erosion marks on the bark is uniform. Debris flows initiate in typically small catchments of a few square kilometers; sediment transport and deposition processes may impact larger catchments [67,68,69].

In this area, slopes of 6–21° have the highest occurrence of mass-wasting. However, Nery [70] considers that slopes greater than 30° facilitate shallow landslides. The average slope angle among all areas suggested that a common threshold controlled the occurrence of landslides [71]. Different areas have different threshold-controlled slope angles that have a relationship with occurrence of mass-wasting hazards. Surface water can rapidly collect in hollows and depressions. Generally, it increases soil moisture, which increases erosion and decreases soil strength. In general, both FR and IV values decrease with the SPI and TW values. The TWI indicates the spatial distribution of humidity conditions. If the soil moisture is high, the possibility that mass-wasting will occur is also high. In the study area, the area formed with gneiss is more likely to suffer hazards. Quaternary sediments and alluvium, flood-plain deposits and terraces, and gravel fans all are made up of immature peddles. Gravel loses the matrix of mud and clay. Softer, highly sheared rocks and mélange areas are more prone to mass-wasting, whereas the various volcanic rocks form somewhat more stable slopes [72]. Van Beek [73] states that agriculture land and steeper slopes show a decrease in temporal activity of land slips, and consequently a decrease in sediment delivery, silting-up, and flooding on these catchments. However, the land use results show a high possibility of mass-wasting hazard occurrence on farmland, which has an impact on local residents in this study, because farmland is mostly located at a low channel elevation (Figure 4) and in the form of terraces on hillsides. The mass-wasting in farmland is not related to large-scale or shallow landslides, but broken farmland terrace dams. The strength of these terraces dams are not very high, which should be paid sufficient attention [74]. It can be seen in Figure 7 and Figure 8 that the channels of the catchments are divided into areas with very high and high susceptibility, and these are areas prone to damage.

The results show that FR performs better than IV. Their AUCs are very close, with the frequency ratio method being slightly more accurate and more applicable than the information value method for defining mass-wasting susceptibility classes. Mass-wasting maps developed using FR and IVM methods may help planners and policy-makers to select appropriate mitigation measures [75]. Researchers have explained that the FR can be used as a supporting method to determine the importance sequence of factors in modeling [76]. However, because the AUC values are close, it cannot be definitively concluded that one model should be selected over the other. The approach to mass-wasting susceptibility mapping should be applicable for a specific area. There is no consensus on the general guidelines for selecting mass-wasting susceptibility influencing factors. Therefore, in this study, the selection and the number of types of mass-wasting susceptibility factors were determined from the characteristics of the geological environment in the four catchments. The influence factors to include in a study should be characteristic of the study area and should be easily selected. Thus, it is worth trying different combinations of influence factors in future works, with the hope that more practical and precise susceptibility maps for mass-wasting management can be achieved.

## 6. Conclusions

Mass-wasting susceptibility mapping focuses on the susceptibility of environmental health impacts and the potential hazards that could affect human health. It may help in providing early warning of environmental health hazards, as well as encouraging emergency preparedness.

Susceptibility surveying and mapping is a major component of the study of the risk and management of mass-wasting hazards. The main objective is to provide decision-makers with a reasonable platform that illustrates the current situation of the study area. The main findings are as follows: (i) field surveys confirmed landslides, collapses, and erosion, which were regarded as mass-wasting hazards. Farmland terraces are mainly located at the bottom of catchments and partly on hillsides. The mass-wasting in farmland mainly relates to broken farmland terrace dams; (ii) according to the FR and IV models, highly susceptible hazard areas in the study area have an elevation of 691–846 m a.s.l., a 0–21° slope angle, flat and convex plan curvatures, an SPI of 8.99–11.12 and 15.1–17.97, a TWI of 3.03–3.43, gneiss rocks, farmland and forest land uses, cinnamon soil, and higher flow accumulation; (iii) different areas have different threshold-controlled slope angles that have a relationship with occurrence of mass-wasting hazards. Softer, highly sheared rocks and mélange areas are more prone to mass-wasting, whereas the various volcanic rocks form stable slopes; (iv) both the FR and IV methods can be used to simulate mass-wasting susceptibility maps, but the FR model has better results in this study. However, because the two methods have close AUC values, both models are useful tools for the estimation of mass-wasting areas to mitigate the devastating impact of mass-wasting hazards.

As a final conclusion, the mass-wasting susceptibility maps produced in this study can be a useful means by which local agencies and decision-makers can plan sustainable and appropriate land use programs and implement development plans. Considering that there are many farmland terraces in the mountainous area in Beijing, especially intermediate- and low-elevation areas, the maps could be useful in an appropriate mass-wasting hazard management plan.

## Figures and Tables

**Table 1:** A list of data sources of each influencing factor.

Influence Factors	Data Sources
Elevation	Generated using GIS from a digital elevation model with a resolution of 5 m
Slope angle	
Curvature	
Stream power index	
Topographic wetness index Flow accumulation	
Lithology	Obtained from a geological map with a scale of 1:10,000
Land use	Google Earth image on May 3, 2014, and field survey
Soil type	Distribution of soil type map in Miyun County with a scale of 1:10,000

**Table 2:** Distribution of the training pixels.

Parameter	Subclass	Mass-Wasting did not Occur		Mass-Wasting Occurred		Total Count	FR	IVM
		Count	Ratio (%)	Count	Ratio (%)			
Elevation (m)	610–691	36,676	9.00	3	6	36,679	0.667	−0.405
	691–749	60,610	14.87	16	32	60,626	2.152	0.766
	749–799	69,605	17.08	11	22	69,616	1.288	0.253
	799–846	69,408	17.03	11	22	69,419	1.292	0.256
	846–891	56,444	13.85	5	10	56,449	0.722	−0.325
	891–935	45,554	11.17	3	6	45,557	0.537	−0.622
	935–980	34,307	8.41	0	0	34,307	0.000	−1.000
	980–1030	20,916	5.13	0	0	20,916	0.000	−1.000
	1030–1103	10,049	2.47	1	2	10,050	0.811	−0.209
	1103–1280	4070	1.00	0	0	4070	0.000	−1.000
Slope angle (°)	0–6	192,418	7.64	6	12	31,160	1.570	0.451
	6–14	28,252	8.43	7	14	34,386	1.660	0.507
	14–21	186,969	9.16	12	24	37,324	2.622	0.964
	21–27	192,418	12.55	7	14	51,185	1.115	0.109
	27–31	28,252	16.58	9	18	67,590	1.086	0.082
	31–35	186,969	17.15	4	8	69,915	0.467	−0.763
	35–39	192,418	14.19	3	6	57,838	0.423	−0.861
	39–44	28,252	9.16	1	2	37,329	0.218	−1.521
	44–50	186,969	4.19	1	2	17,075	0.478	−0.739
	50–73	192,418	0.95	0	0	3887	0.000	−1.000
Curvature	Concave	192,418	47.21	32	64	192,450	1.356	0.304
	Flat	28,252	6.93	6	12	28,258	1.731	0.549
	Convex	186,969	45.86	12	24	186,981	0.523	−0.648
SPI	0–2.57	65,867	16.16	3	6	65,870	0.371	−0.991
	2.57–6.21	169,540	41.59	12	24	169,552	0.577	−0.550
	6.21–8.99	90,167	22.12	15	30	90,182	1.356	0.305
	8.99–11.12	35,453	8.70	13	26	35,466	2.989	1.095
	11.12–13.05	17,690	4.34	2	4	17,692	0.922	−0.082
	13.05–15.19	6509	1.60	1	2	6510	1.253	0.225
	15.19–17.97	3284	0.81	1	2	3285	2.482	0.909
	17.97–22.04	1300	0.32	0	0	1300	0.000	−1.000
	22.04–29.52	265	0.07	0	0	265	0.000	−1.000
	29.52–54.55	17,564	4.31	3	6	17,567	1.393	0.331
TWI	0–3.03	43,441	10.66	7	14	43,448	1.314	0.273
	3.03–3.43	57,290	14.06	18	36	57,308	2.561	0.940
	3.43–3.97	78,098	19.16	10	20	78,108	1.044	0.043
	3.97–4.58	89,843	22.04	9	18	89,852	0.817	−0.203
	4.56–5.32	68,776	16.87	3	6	68,779	0.356	−1.034
	5.32–6.33	41,754	10.24	2	4	41,756	0.391	−0.940
	6.37–7.56	19,873	4.87	0	0	19,873	0.000	−1.000
	7.56–9.38	6523	1.60	1	2	6524	1.250	0.223
	9.38–14.44	1768	0.43	0	0	1768	0.000	−1.000
	14.44–18.83	273	0.07	0	0	273	0.000	−1.000
Lithology	Gneiss	65,480	16.06	7	14	65,493	1.619	0.482
	Quartzite	199,298	48.89	18	36	199,324	1.064	0.062
	Diorite	91,377	22.42	10	20	91,387	0.892	−0.114
	Acid rock	29,299	7.19	9	18	29,300	0.278	−1.279
	Granite	22,143	5.43	3	6	22,143	0.000	−1.000
Land use	Construction	8367	2.05	0	0	8373	0.000	−1.000
	Farmland	26,505	6.50	6	12	26,505	1.846	0.613
	Forest	372,768	91.45	44	88	372,812	0.962	−0.038
Soil type	Cinnamon soil	166,659	40.88	23	46	166,682	1.125	0.118
	Brown soil	240,979	59.12	27	54	241,006	0.914	−0.091
Flow accumulation	0–2442	57,465	14.10	5	10	57,470	0.709	−0.343
	2442–4884	46,594	11.43	3	6	46,597	0.525	−0.644
	4884–9768	71,240	17.48	7	14	71,247	0.801	−0.222
	9768–14,652	51,253	12.57	6	12	51,259	0.954	−0.047
	14,652–26,862	51,073	12.53	3	6	51,076	0.479	−0.736
	26,862–46,398	38,437	9.43	6	12	38,443	1.273	0.241
	46,398–117,217	34,695	8.51	3	6	34,698	0.705	−0.350
	117,217–351,651	27,961	6.86	5	10	27,966	1.458	0.377
	351,651–622,719	28,921	7.10	12	24	28,933	3.382	1.218

Note: FR: frequency ratio; IVM: information value model; SPI: the stream power index; TWI: the topographic wetness index.

**Table 3:** Mass-wasting susceptibility mapping results in the study area.

Class	FR			IVM		
	Number of Grids	Area (km^2^)	Ratio (%)	Number of Grids	Area (km^2^)	Ratio (%)
Very Low	99,911	2.50	24.55	70,450	1.76	17.31
Low	133,203	3.34	32.74	111,088	2.78	27.30
Moderate	89,531	2.24	22.00	95,677	2.40	23.51
High	56,350	1.41	13.85	80,126	2.01	19.69
Very high	27,918	0.70	6.86	49,572	1.24	12.18

Note: FR: frequency ratio; IVM: information value model.
